# A regenerative metal–organic framework for reversible uptake of Cd(ii): from effective adsorption to *in situ* detection†
†Electronic supplementary information (ESI) available: Additional details of experimental procedure, PXRD, additional figures for **FJI-H9** and **FJI-H10**. CCDC 1047502 and 1047503. For ESI and crystallographic data in CIF or other electronic format see DOI: 10.1039/c6sc00972g


**DOI:** 10.1039/c6sc00972g

**Published:** 2016-05-25

**Authors:** Hui Xue, Qihui Chen, Feilong Jiang, Daqiang Yuan, Guangxun Lv, Linfeng Liang, Luyao Liu, Maochun Hong

**Affiliations:** a State Key Lab of Structure Chemistry , Fujian Institute of Research on the Structure of Matter , Chinese Academy of Sciences , Fuzhou , Fujian 350002 , China . Email: hmc@fjirsm.ac.cn ; Email: chenqh@fjirsm.ac.cn ; Fax: +86-591-83794946 ; Tel: +86-591-83792460; b University of the Chinese Academy of Sciences , Beijing , 100049 , China

## Abstract

An extraordinary metal–organic framework **FJI-H9** with high adsorption and selectivity for the reversible uptake of Cd(ii) has been developed. Further research indicates that such high absorption results from an unusual synergy from active sites and the confined cavity. In addition, fast detection of Cd(ii) at low concentrations down to 10 ppm and *in situ* reconstruction of the used framework into a fresh one have also been successfully achieved.

## Abstract 2

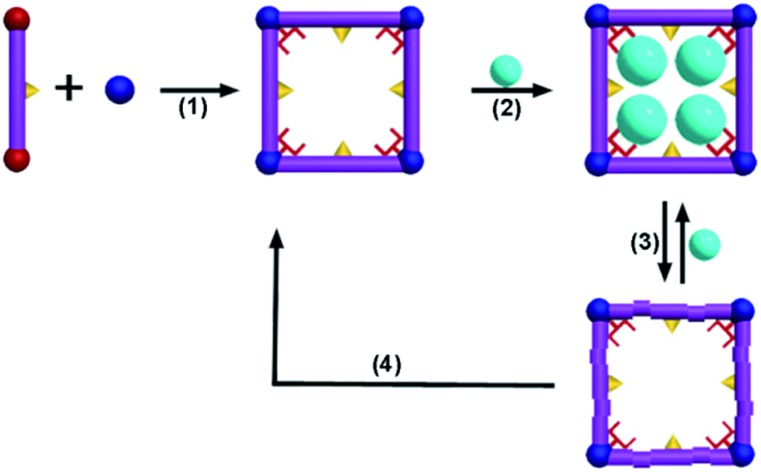
A metal–organic framework with high adsorption and selectivity for reversible uptake of Cd(ii) was developed. Both fast detection of Cd(ii) at low concentration and *in situ* reconstruction of the used MOF were successfully achieved.

## Introduction

Heavy metal pollution which can cause brain damage and disease in humans and other species has become a serious threat to environment. For example, cadmium is known to directly damage the nervous, reproductive, renal and skeletal systems, and can also cause some cancers.^1^ Therefore, how to remove cadmium effectively from industrial processes is highly urgent. Many sorbent materials, such as carbon materials,^2^ biomass,^3^ nanoparticles^4^ and chelating polymers^5^ have been extensively investigated for the adsorptive removal of cadmium; however those sorbents face several challenges, such as low uptake and moderate affinity for Cd(ii).

**Scheme 1:**
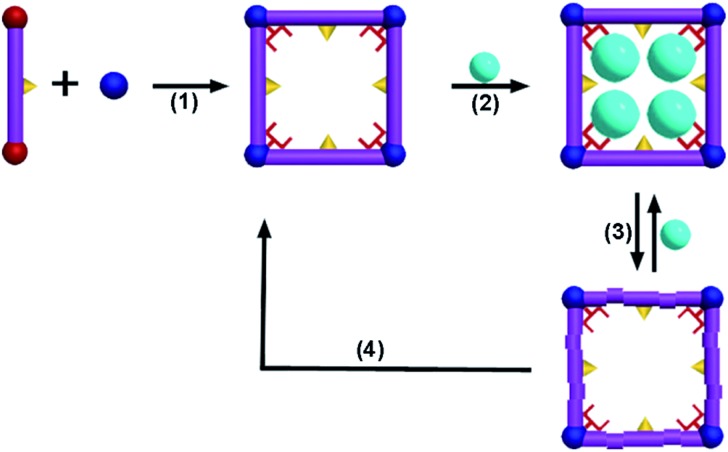
A regenerative MOF for reversible uptake of Cd(ii): (1) self-assembly 3D framework from organic ligand and hard metal ion; (2) effective absorption with high capacity; (3) absorption and desorption; (4) reconstruction of the used sample into a fresh sample.

Metal–organic frameworks (MOFs) are a new type porous material composed of organic bridging ligands and metal ions or metal ion clusters, possessing high porosities and ready tenability.^6^ Many applications based on MOFs including gas storage,^7^ separation^8^ and catalysis^9^ have been developed. Recently, MOFs have been used as solid-phase sorbents to remove heavy metals by the introduction of suitable functional groups.^10^ So far, many excellent sorbents for Hg(ii) based on MOFs or COFs have been developed based on strong Hg–S interactions.10a,e,i However, the exploration of MOFs for Cd(ii) removal is highly challenging, and only two examples with moderate capacity have been reported.10m,j Herein, a novel metal–organic framework **FJI-H9** for the reversible uptake of Cd(ii) has been developed, and the adsorption of Cd(ii) achieved is up to 286 mg g^–1^. Furthermore, fast detection of Cd(ii) at low concentrations down to 10 ppm and the *in situ* reconstruction of the used framework into a fresh one have also been successfully carried out (Scheme 1).

## Results and discussion

### Syntheses and structures of **FJI-H9**

**Fig. 1:**
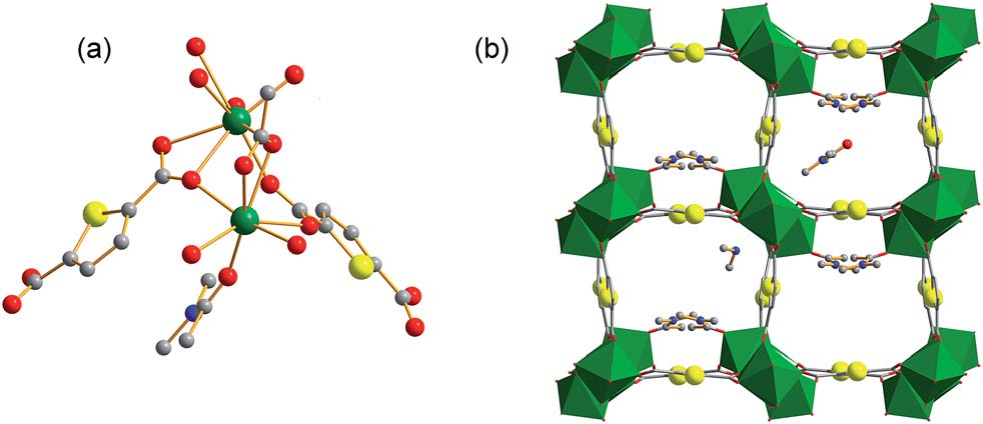
(a) The coordination environment of **FJI-H9**, (C = grey, N = blue, O = red, S = yellow, Ca = green, H was omitted for clarity); (b) the 3D framework of **FJI-H9** (sulfur atoms are highlighted as yellow balls, Ca atoms are highlighted as green polyhedra).

Using organic donors composed of carboxylic and thiophenol with hard metal ions has proved to be a powerful strategy in the construction of porous frameworks for the adsorption of Hg(ii).10*e* So we tried to react 2,5-thiophenedicarboxylate (H_2_thb), composed of two carboxylic groups and one thiophenyl, with hard metal ions to obtain a MOF framework for the adsorption of Cd(ii). Herein, a novel MOF **FJI-H9**, formulated as [Me_2_NH_2_^+^][Ca_2_(thb^2–^)_2_(CH_3_COO^–^)(DMA)]·DMA has been synthesized by reacting CaCl_2_ with 2,5-thiophenedicarboxylate. Single crystal X-ray analysis reveals that **FJI-H9** crystallizes in the orthorhombic space group *Pca*2_1_, its asymmetric unit contains two calcium ions, two thb^2–^ ligands, one coordinated CH_3_COO^–^ and a DMA molecule, as well as one dissociative DMA molecule and one [Me_2_NH_2_]^+^ ion. The Ca(1) atom is coordinated by seven oxygen atoms, in which four oxygen atoms from four thb^2–^ ligands, two oxygen atoms from one CH_3_COO^–^ molecule and one oxygen atom from the DMA molecule (Ca–O, 2.292–2.647 Å). Meanwhile the Ca(2) atom is coordinated by eight oxygen atoms, with six oxygen atoms from four thb^2–^ molecules and two oxygen atoms from two CH_3_COO^–^ molecules (Ca–O, 2.292–2.647 Å). As shown in Fig. 1b, a series of 1D channels (11.349 × 11.235 Å) based on Ca(ii) and the ligand array along the *a* axis in the framework of **FJI-H9**, in which sulfur-functions highlighted as yellow balls remain free-standing, both the coordinated DMA molecules and free DMA molecules stack in its cavity orderly .

### Heavy metal ion adsorption test of **FJI-H9**

**Fig. 2:**
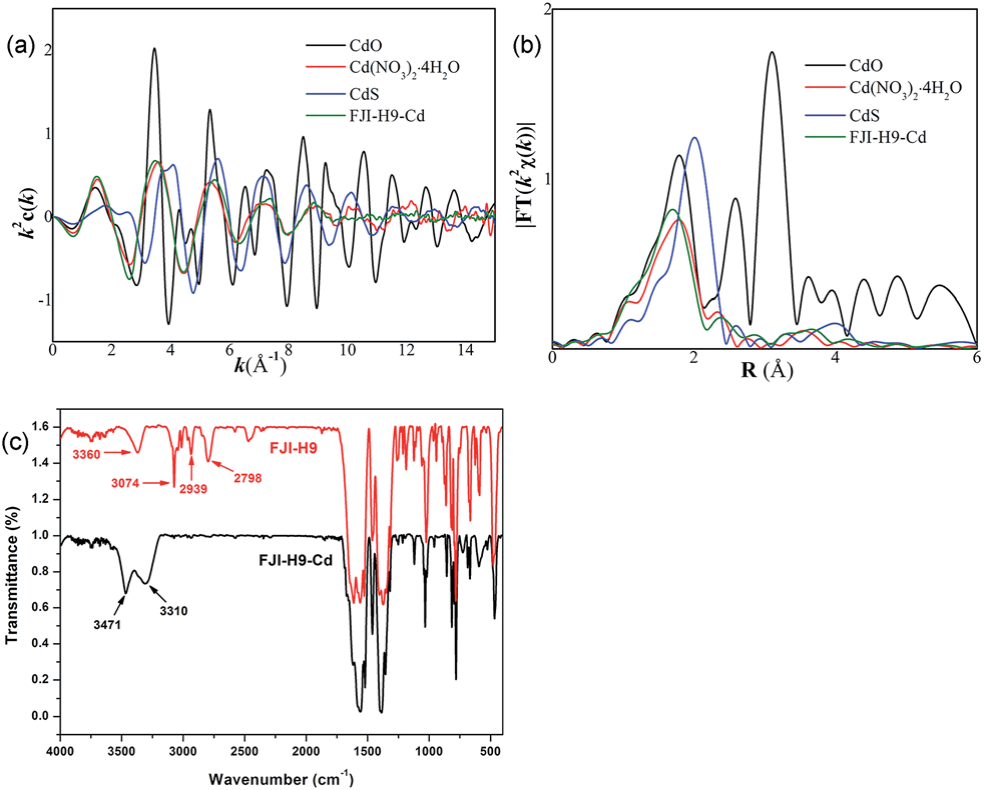
(a) Cd K-edge extended XAFS oscillation function *k*^2^*χ*(*k*); (b) the corresponding Fourier transforms FT(*k*^3^*w*(*k*)); (c) the IR spectrum of **FJI-H9** and **FJI-H9-Cd** (red = **FJI-H9**, black = **FJI-H9-Cd**), *V*_s_ = 3360 cm^–1^ belongs to NH, *V*_s_ = 3074 cm^–1^, 2939 cm^–1^, 2798 cm^–1^ belong to CH_3_ from [Me_2_NH_2_]^+^ and DMA molecules.

**Scheme 2:**
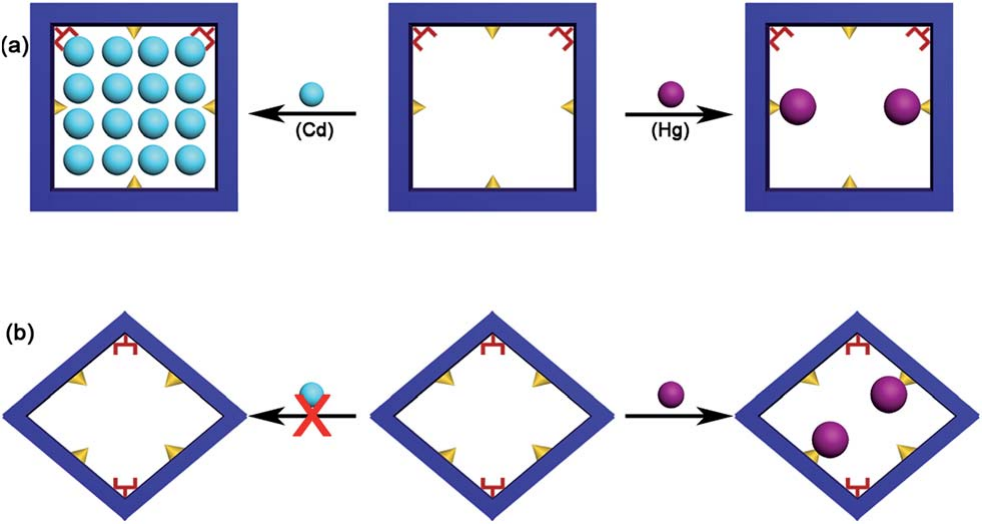
Possible adsorption mechanisms: (a) with help by DMA molecules highlighted as red fork and confined cavity, the Cd(ii) ions close-pack in the square channel of **FJI-H9**; only part of the thiophenyl groups highlighted as yellow triangles from **FJI-H9** adsorb Hg(ii). (b) Nearly no Cd(ii) is absorbed by **FJI-H10**, only part of the thiophenyl groups highlighted as yellow triangles from **FJI-H10** adsorb Hg(ii).

Both the free and coordinated DMA molecules are occupied in the channels of **FJI-H9**, which may highly improve the adsorption of Cd(ii). Thus, fresh **FJI-H9** without activation has been used to adsorb heavy metal ions directly. The as-made fresh crystals of **FJI-H9** (28 mg) were directly soaked in 25 mL of a 0.1 mol L^–1^ acetonitrile solution of Cd(NO_3_)_2_. The solid was then isolated further and washed with acetonitrile to remove the residual Cd(NO_3_)_2_ on the exterior of the **FJI-H9** powder. The solid sample thus obtained (denoted as **FJI-H9-Cd**) was subjected to regular CHN, inductively coupled plasma (ICP) elemental and thermogravimetry-mass spectrometry analyses. ICP analysis determined the Cd/Ca ratio to be 2.5 : 1 (Table 1), TG-MS analysis demonstrates that there are about 15.5% volatile solvents which contain H_2_O and DMA molecules. All of these lead to a composition of (Cd^2+^)_0.5_Ca_2_(tda^2–^)_2_(CH_3_COO^–^)(DMA)_1.2_·[Cd(NO_3_)_2_]_1.28_·(H_2_O)_7.1_, (calculated: [C (22.87%), H (3.10%), N (5.66%)]; found: [C (22.95%), H (3.38%), N (5.35%)]). This demonstrates that 1 mol **FJI-H9** can adsorb 1.78 mol Cd(ii), and the adsorption of Cd(ii) is up to 286 mg g^–1^. However, the saturated adsorption based on the adsorption isotherm is more practical in the application of decontaminating heavy metal ions; so adsorption isotherm for Cd(ii) removal from acetonitrile was carried out, and the maximum Cd(ii) adsorption capacity of **FJI-H9** was found to be about 225 mg g^–1^ (ESI, Fig. S5†). Further research demonstrated that only 0.13 mol Hg are absorbed by per mol **FJI-H9**. In contrast, other metal ions such as Mg(ii), Co(ii), Ni(ii), Mn(ii), Zn(ii), Fe(ii) and Pb(ii) cannot be absorbed by **FJI-H9**. The activation sites for the adsorption of Hg(ii) may be these thiophenyl groups, so Hg is only moderately absorbed. It is highly interesting that Cd(ii) can be highly adsorbed while Hg(ii) is only moderately adsorbed. In order to further investigate the Cd(ii) absorption character of **FJI-H9**, many other necessary tests such as extended X-ray absorption fine structure (EXAFS) and infrared radiation (IR) have been carried out (Fig. 2). EXAFS analysis shows that there is no Cd–S interaction in the **FJI-H9-Cd** powder, indicating that the Cd atoms do not bond with S atoms from the thb^2–^ ligands (Fig. 2a). Further quantitative EXAFS curve-fitting demonstrates that there are three different kinds of Cd–O interactions around Cd atoms in the **FJI-H9-Cd** powder, including four Cd–O_1_ (*R* = 2.26 Å), two Cd–O_2_ (*R* = 2.39 Å) and two Cd–O_3_ (*R* = 2.54 Å) (Table 2). IR analysis implies that the strong N–H (*V*_s_ = 3360 cm^–1^) spectra from [Me_2_NH_2_]^+^ ion disappears while another strong hydrogen interaction (*V*_s_ = 3310 cm^–1^, 3471 cm^–1^) is formed. So the high adsorption of Cd(ii) may result from the following: (1) electronegative framework is beneficial to adsorption of Cd(ii), where isolated [Me_2_NH_2_]^+^ can be replaced by Cd^2+^, which can be confirmed by the EA analysis and IR analysis (as shown in Fig. 2, the characteristic spectrum of [Me_2_NH_2_]^+^ ion has disappeared); (2) this-sized square cavity of **FJI-H9** may be very suitable for the closing pack of Cd(ii), and a suitable cavity may play the most important role for the adsorption of metal ions here. Both the EXAFS analysis [four Cd–O_1_ (*R* = 2.26 Å)] and EA analysis (the ration of Cd/H_2_O = 1 : 4) imply the existence of Cd(H_2_O)_4_^2+^. Another two different Cd–O interactions [Cd–O_2_ (*R* = 2.39 Å), Cd–O_3_ (*R* = 2.54 Å)] may indicate that NO_3_^–^ anions are stacked around Cd(H_2_O)_4_^2+^ closely, which is also confirmed by the new-formed strong hydrogen interaction (*V*_s_ = 3310 cm^–1^, 3471 cm^–1^). (3) The activated sites for adsorbing Cd(ii) may be these coordinated DMA molecules rather than the thiophenyl, in which the Cd(H_2_O)_4_^2+^ ions are immobilized by DMA molecules through strong hydrogen bond. In summary, the high adsorption of Cd(ii) of **FJI-H9** results from an unusual synergy from the active sites and confined cavity in Scheme 2(a).

**Table 1:** The weight ratios of M/Ca in **FJI-H9**

	Cd^2+^	Hg^2+^	Ni^2+^	Mn^2+^	Zn^2+^	Fe^2+^	Pb^2+^	Co^2+^	Mg^2+^
M/Ca	2.50	0.34	0.08	0.07	0.06	0.07	0.009	0.07	0.03

**Table 2:** Structural parameters extracted from quantitative EXAFS curve-fitting

Sample	Path	*N*	*R* (Å)	*σ* ^2^ (10^–3^ Å^2^)	Δ*E*_0_ (eV)
CdO	Cd–O	6.0	2.34	3.8	3.0
CdS	Cd–S	4.0	2.52	3.3	4.8
	Cd–O_1_	4.0	2.31	5.6	5.0
Cd(NO_3_)_2_·4H_2_O	Cd–O_2_	2.0	2.45	6.2	5.0
	Cd–O_3_	2.0	2.59	7.5	5.0
	Cd–O_1_	4.0	2.26	4.8	3.8
**FJI-H9-Cd**	Cd–O_2_	2.0	2.39	5.3	3.8
	Cd–O_3_	2.0	2.54	6.5	3.8

### The selective adsorption of **FJI-H9** under background metal ions

**Fig. 3:**
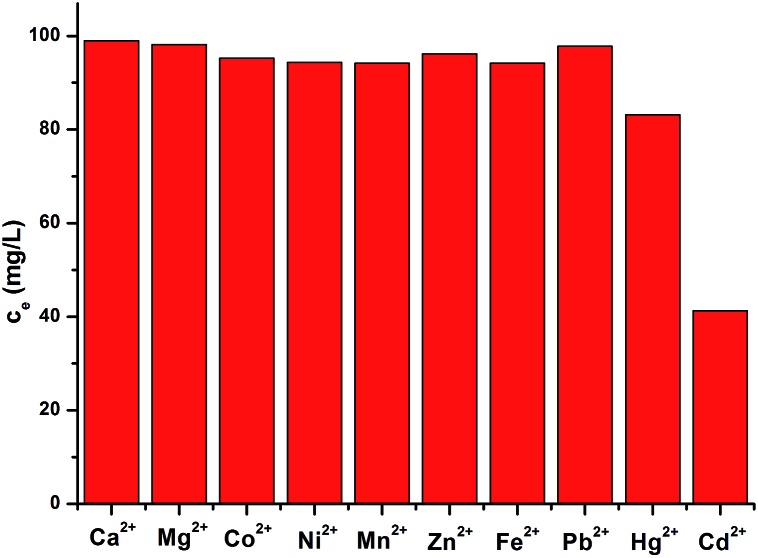
The selective adsorption of Cd(ii) by **FJI-H9**.

Selectivity tests were also performed on **FJI-H9** in a mixed solution containing Cd(ii), Hg(ii), Ca(ii), Mg(ii), Co(ii), Ni(ii), Mn(ii), Zn(ii), Fe(ii) and Pb(ii) ions. As shown in Fig. 3, **FJI-H9** can remain effective in the presence of high concentrations of these background metal ions, Cd(ii) ions can be effectively adsorbed while Hg(ii) can be moderately adsorbed. In contrast, other background metal ions such as Ca(ii), Mg(ii), Co(ii), Ni(ii), Mn(ii), Zn(ii), Fe(ii) and Pb(ii) do not quite bind to **FJI-H9**.

**Fig. 4:**
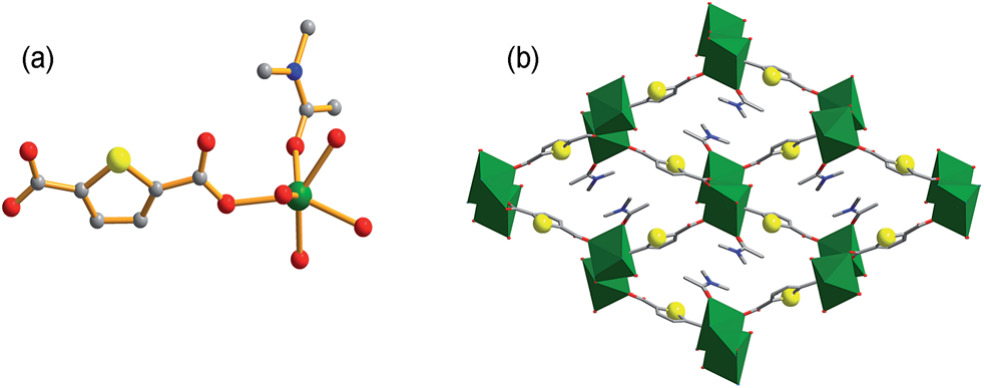
(a) Coordination environment of **FJI-H10**, (C = grey, N = blue, O = red, S = yellow, Ca = green, H was omitted for clarity); (b) the 3D framework of **FJI-H10** (sulfur atoms highlighted as yellow balls, Ca atoms highlighted as green polyhedra).

In order to prove the importance of the suitable cavity for adsorption of Cd(ii), another framework **FJI-H10** formulated as [Ca(thb^2–^)(DMA)(H_2_O)] was also prepared from CaCl_2_ and 2,5-thiophenedicarboxylate. Single crystal X-ray analysis indicates that **FJI-H10** crystallizes in the monoclinic space group *P*2_1_/*n*, and the asymmetric unit contains one calcium atom, one thb^2–^ ligand, one coordinated DMA and H_2_O molecule. The Ca(ii) atom is coordinated by six oxygen atoms and exhibits a distorted octahedron coordination geometry, with four oxygen atoms from four different thb^2–^ ligands, one oxygen atom from a H_2_O molecule and one oxygen atom from a DMA molecule (Ca–O, 2.289–2.434 Å). Compared with **FJI-H9**, **FJI-H10** also has coordinated DMA molecules and free thiophenyl, herein the cavity is rhombic (11.744 × 11.648 Å) rather than square (Fig. 4). Further research demonstrates that about 0.11 mol Hg(ii) are absorbed by per mol **FJI-H10**, and nearly no Cd(ii) can be absorbed by **FJI-H10** (Table S4,† Scheme 2b). Moderate adsorption of Hg(ii) from **FJI-H10** also may result from thiophenyl groups. It is highly interesting that nearly no Cd(ii) can be captured by **FJI-H10**, which may further prove that a high adsorption of Cd(ii) by **FJI-H9** indeed results from an unusual synergy from active sites and a confined cavity (Table 3).

**Table 3:** Comparison of cadmium adsorption performances by various adsorbents

Sorbent	Adsorption capacity (mg g^–1^)
**FJI-H9** (MOF)	286^*a*^/225^*b*^
Mg-MTMS5*e*	210^*b*^
Manganese dioxide formed *in situ*4*d*	176^*b*^
PCN-100 (ref. 10*m*) (MOF)	147^*a*^
ZrO_2_/B_2_O_3_ (ref. 4*c*)	109.9^*b*^
Cu_3_(BTC)_2_-SO_3_H10*j* (MOF)	88.7^*b*^
MPGI5*d*	87.7^*b*^
Sulfonic-functionalized poly(dimethylsiloxane) networks5*a*	78.7^*b*^
Magnetic yeast treated with EDTA dianhydride4*b*	48.7^*b*^
Salicylic acid type chelate5*b*	45.0^*b*^
PPBM^3^	43.5^*b*^
Si-DTC5*c*	40.5^*b*^
Fe_3_O_4_@APS@AA-*co*-CA4*a*	29.6^*b*^

^*a*^Directly obtain from the solid ICP test.

^*b*^Obtain from the adsorption isotherm.

### Desorption and resorption of Cd(NO_3_)_2_ on **FJI-H9**

**Scheme 3:**
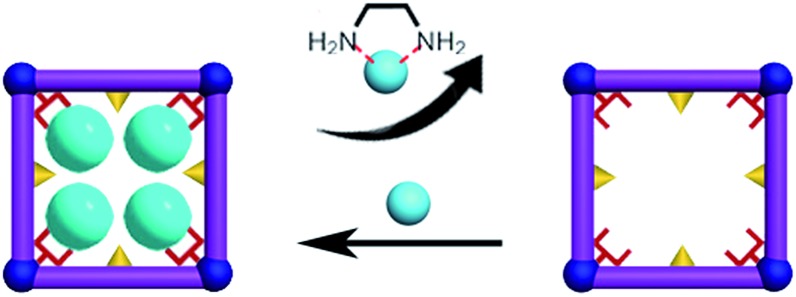
Desorption and resorption of Cd(NO_3_)_2_ on **FJI-H9**.

**Fig. 5:**
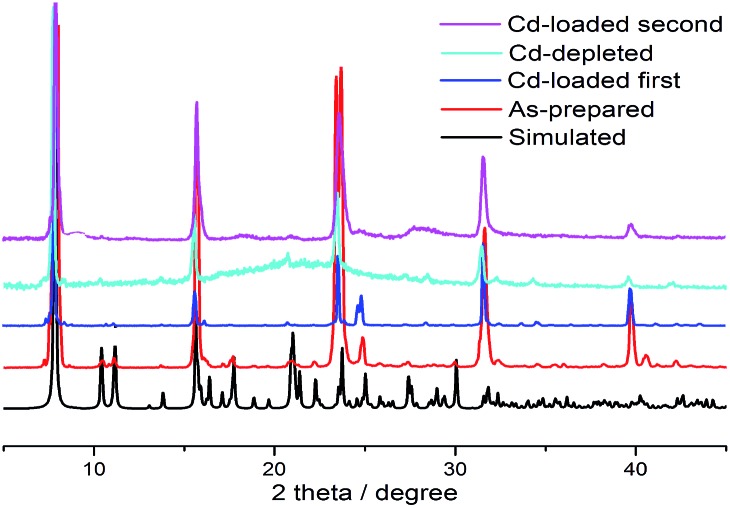
Powder X-ray diffraction analyses. Black: a simulation sample of **FJI-H9**; red: a synthesized sample of **FJI-H9**; blue: a first Cd(NO_3_)_2_-loaded sample of **FJI-H9**; light blue: a Cd(NO_3_)_2_-depleted sample of **FJI-H9-Cd**; purple: a second Cd(NO_3_)_2_-loaded sample of **FJI-H9**.

**FJI-H9** is an effective adsorbent for Cd(ii), which represents a new record for adsorption of Cd(ii). Can this effective adsorbent be reused? In order to remove the Cd(ii) adsorbed in **FJI-H9-Cd** powder, ethylenediamine (EDA), which has more strong interactions with Cd(ii), has been selected (Scheme 3). Further researches indicate that about 99.9% of Cd(NO_3_)_2_ can be removed from **FJI-H9-Cd** powder with the help of EDA (ESI†). The used **FJI-H9** framework was re-used to adsorb Cd(NO_3_)_2_ once again, and the ICP reveals the weight ratio of Cd/Ca reduce to 2.03 : 1, indicating that the efficiency of **FJI-H9** was little reduced after multiple usages. Powder XRD analyses demonstrate that the main framework of **FJI-H9** remains stable after loading and reloading (Fig. 5).

### Reconstruction of the used **FJI-H9**

**Scheme 4:**
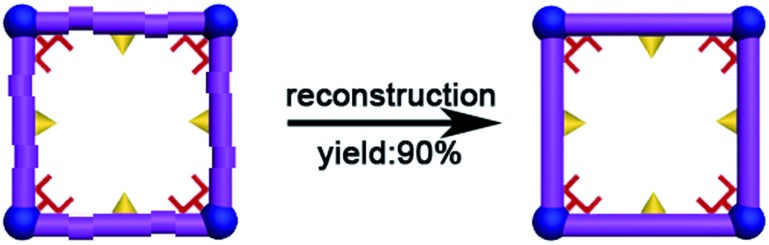
*In situ* reconstruction of the used framework into fresh framework.

Although **FJI-H9** is reusable, its adsorption capacity is reduced after multiple usages. Can the used **FJI-H9** be reconstructed into a fresh one? If so, it will greatly reduce the costs and improve efficiency. Based on the reversibility of the coordination bond, reconstruction of the used framework into a fresh framework is possible. After many attempts, we finally find that the used **FJI-H9** powder can be *in situ* reconstructed into a fresh **FJI-H9** crystal as follows: the 10 mg used **FJI-H9** powder and 50 uL HNO_3_ (about 16 mol L^–1^) were added to 5 mL mother liquor which had been used to prepare fresh **FJI-H9**. Heating such a solution at 85 °C for six days leads to 9 mg of fresh **FJI-H9** crystals. The cost of remove Cd(ii) is highly reduced through the effective regeneration of **FJI-H9**, which may provide a new strategy to develop more economical adsorbents for heavy metal ions. As far as we know, reconstruction of used adsorbents into fresh ones has not been reported until now (Scheme 4).

### Fluorescence indicator

**Fig. 6:**
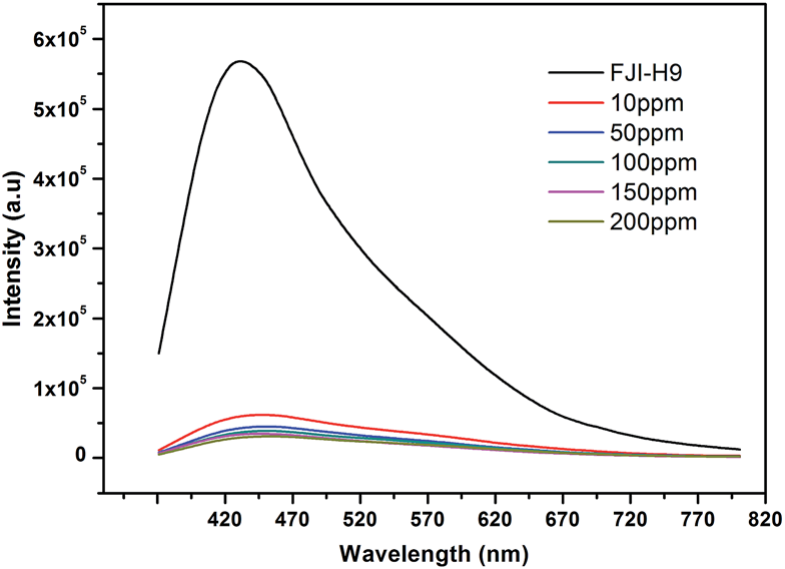
Rapid detection of cadmium with low concentration.

The literature shows that MOFs as heavy metal adsorbents also can effectively detect heavy metal ions, but such a process usually needs long times or high concentrations of heavy metal ions.^10^ However, the industrial cadmium pollution level is only 10 ppm in general, so a more effective indicator for heavy metal ions should be developed. We have found that **FJI-H9** is a good adsorbent for Cd(ii), and it also can be reused and *in situ* regenerated. Can such a material *in situ* detect Cd(ii) more effectively and quickly? For this purpose, **FJI-H9** was added to a Cd(NO_3_)_2_ solution of different concentration. As shown in Fig. 6, the intensity slightly reduced when the concentration gradually increased from 10 ppm to 200 ppm. Interestingly, even soaking **FJI-H9** in the 10 ppm solution of Cd(ii) for 10 minutes can also highly quench its emission (*λ*_em_ = 460 nm), in which 92% of the total fluorescence intensity of **FJI-H9** can be quenched. The quenched fluorescence in Fig. 6 likely originates from the interaction between the coordinated DMA molecules and Cd(ii) ions. Such quick and effective fluorescence quenching may result from the fact that so many coordinated DMA molecules and free DMA molecules are stacked in the 1D channels of the **FJI-H9** framework, which make intermolecular energy transfer between the **FJI-H9** framework and Cd(ii) ions more easily. In contrast, for other metal ions such as Hg(ii), Mg(ii), Co(ii), Ni(ii), Mn(ii), Zn(ii), Fe(ii) and Pb(ii), no such obvious fluorescence quenching can be observed under the same conditions. To the best of our knowledge, using MOFs to detect Cd(ii) ions in such an effective way has not been reported previously.

## Conclusions

An exceptional MOF **FJI-H9** for reversible uptake of Cd(ii) with high absorption and selectivity has been obtained successfully, and *in situ* reconstruction of the used **FJI-H9** framework into a fresh one also has been achieved. Furthermore, the quick detection of Cd(ii) at low concentrations down to 10 ppm also has been realized for the first time. All of these characters of **FJI-H9**, which contains high adsorption, regeneration, and quick detection, will provide a new strategy to prepare more effective adsorbents to remove and detect heavy metal ions.

## Supplementary Material

Supplementary information

Crystal structure data
