# Supplementary material for: A regenerative metal–organic framework for reversible uptake of Cd(ii): from effective adsorption to in situ detection
Source: Chem Sci. 2016 May 25;7(9):5983–8. doi: 10.1039/c6sc00972g (PMC6024582; doi:10.1039/c6sc00972g)
Supplement: Supplementary file 1 [file SC-007-C6SC00972G-s001.pdf]

Electronic Supplementary Material (ESI) for Chemical Science.
This journal is © The Royal Society of Chemistry 2016

Supporting information

A regenerative metal-organic framework for reversible uptake of Cd(II): from effective adsorption to *in situ* detection

Hui Xue,[a,b] Qihui Chen,*[a,b] Feilong Jiang,[a] Daqiang Yuan,[a,b] Guangxun Lv,[a,b] Linfeng Liang,[a,b] Luyao Liu,[a] and Maochun Hong*[a,b]

[a]*State Key Laboratory of Structure Chemistry, Fujian Institute of Research on the Structure of Matter, Chinese Academy of Sciences, Fuzhou, Fujian, 350002, China*
[b]*University of the Chinese Academy of Sciences, Beijing, 10049, China*

Content

(1) Synthesis and crystal structures determination of FJI-H9 and FJI-H10.
(2) Table S1 Crystal data and refinement results for FJI-H9 and FJI-H10.
(3) Table S2 Selected Bond Lengths (Å) and Angles (°) for FJI-H9.
(4) Table S3 Selected Bond Lengths (Å) and Angles (°) for FJI-H10.
(5) Table S4 The weight ratios of M/Ca in FJI-H9.
(6) Table S5 The weight ratios of M/Ca in FJI-H10.
(7) Scheme 2 Possible adsorption mechanisms.
(8) Figure S3 TGA curves.
(9) Figure S4 Thermogravimetry-Mass curves.
(10) Figure S5 Sorption isotherm of Cd^{2+} by FJI-H9. (v = 30 mL, m (adsorbent) = 10 mg, T = 20 °C, t = 15 h).
(11) Figure S6 The selective adsorption of Cd^{2+} by FJI-H9.
(12) Figure S7 EXAFS measurements.
(13) Desorption of FJI-H9.
(14) Scheme 3 Desorption and resorption of $Cd(NO_3)_2$ by FJI-H9.
(15) Secondary adsorption of $Cd(NO_3)_2$ test.
(16) Figure S8 Powder X-ray diffraction patterns.
(17) Table S6 The weight ratios of Cd/Ca in FJI-H9 for the first and second times.
(18) Figure S9 Powder X-ray diffraction patterns.
(19) Regeneration used FJI-H9 into fresh one.
(20) Scheme 4 *In situ* reconstruction the used framework into fresh framework.
(21) Fluorescence indicator.
(22) Figure S10 Rapid detection of cadmium with low concentration.

Synthesis of FJI-H9

FJI-H9 was obtained by dissolving $CaCl_2$ (0.1 mmol, 0.011 g) and H_2thb (0.1 mmol, 0.017 g) in DMA/H_2O (v_1:v_2 = 2:1) and 50 uL HNO_3 was added, then heating this solution at 85 °C for three days, then the rodlike crystal obtained in 85% yield. Anal. calcd for $C_{24}H_{33}Ca_2N_3O_{12}S_2$ (Mr = 699.82): C, 41.19; H, 4.72; N, 6.01. Found: C, 40.88; H, 4.70; N, 6.00.

Single crystal X-ray analysis revealed that **FJI-H9** was crystallized in the orthorhombic space group *Pca*2$_1$. The symmetric unit of **FJI-H9** contained two calcium atoms, two thb^{2-} ligands, one coordinated CH_3COO^- and DMA molecule, and one uncoordinated DMA and $Me_2NH_2^+$. Ca1 was seven-coordinated defined by four oxygen atoms from four thb^{2-} ligands, two oxygen atoms from one CH_3COO^- molecule and one oxygen atom from DMA molecule. Ca2 was eight-coordinated completed by six oxygen atoms from four thb^{2-} molecules and two oxygen atoms from two CH_3COO^- molecules. The Ca–O bond lengths were in the range of 2.292–2.647 Å and the Ca1–Ca2 bond length was 3.746 Å.

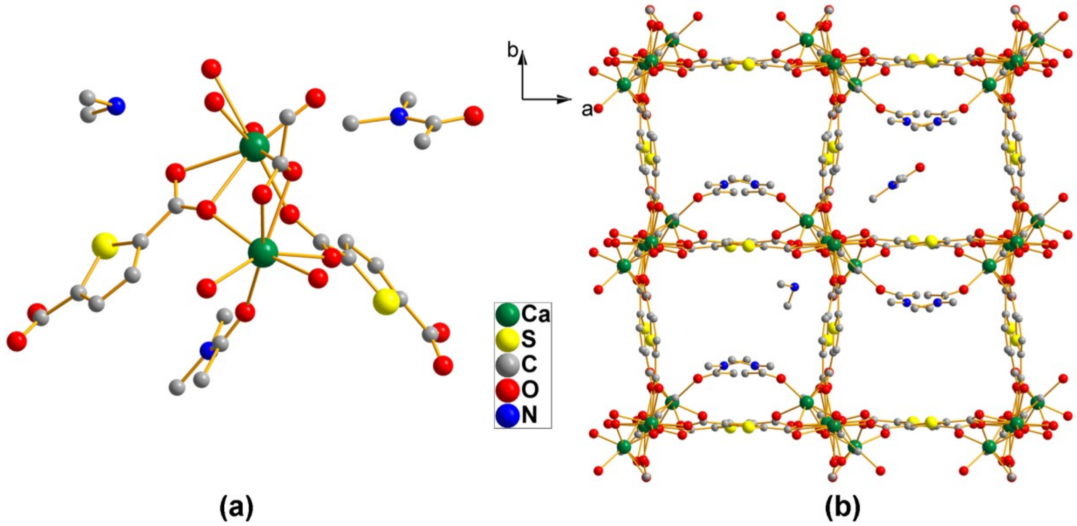

Figure S1 (a) the symmetric unit of **FJI-H9**; (b) the 3D framework of **FJI-H9**.

Synthesis of compound FJI-H10

FJI-H10 was obtained by dissolving $CaCl_2$ (0.1 mmol, 0.011 g) and H_2thb (0.1 mmol, 0.017 g) in DMA/H_2O (v_1:v_2 = 2:1) and heating this solution at 85 ^oC for three days, then the rodlike crystal obtained in 62% yield. Anal. calcd for $C_{10}H_{13}CaNO_6S$ (Mr = 315.36): C, 38.09; H, 4.13; N, 4.44. Found: C, 38.07; H, 4.17; N, 4.21.

X-ray diffraction study indicated that **FJI-H10** belongs to the monoclinic space group $P2_1/n$ and the symmetric unit contained one calcium atom, one organic ligand, one coordinated DMA and H_2O molecule. The Ca(II) atom was coordinated in a distorted octahedral geometry by four O atoms from four different ligands, one O atom from H_2O molecule and one O atom from DMA molecule. The ligand adopted two different kinds of coordination modes through two carboxyl groups. Single O atom of one carboxyl coordinated with one Ca(II) ion while the other O atom did not coordinate. Another carboxyl acted as a bridging linker which coordinated with three Ca(II) ions by two O atoms. The Ca–O bond lengths were in the range of 2.289–2.434 Å.

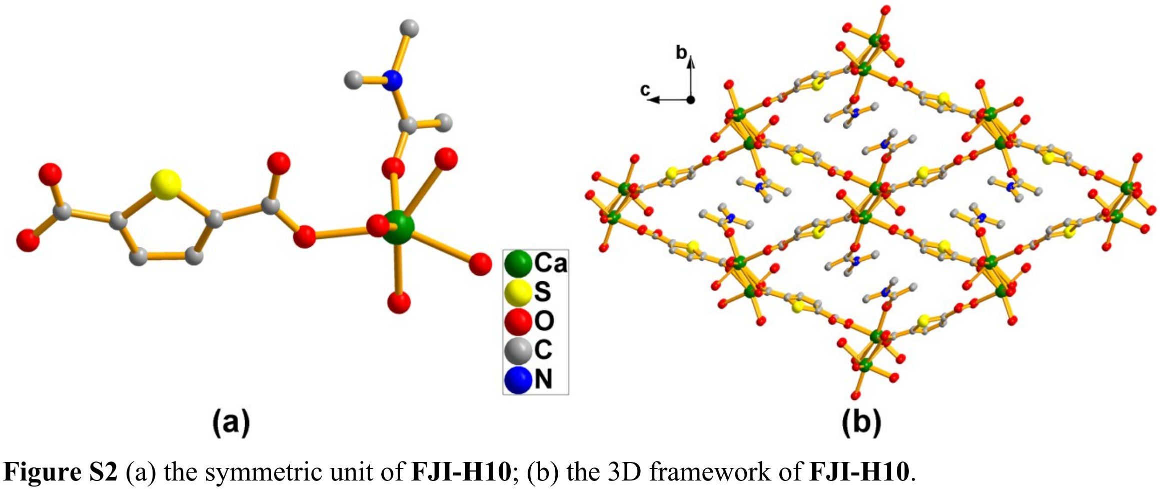

Figure S2 (a) the symmetric unit of **FJI-H10**; (b) the 3D framework of **FJI-H10**.

X-ray crystallography

Data collections were all performed on a Mercury CCD diffractometer with graphite monochromated CuKa radiation (λ = 0.71073 Å). The structures were solved by direct methods, and all calculations were performed using the SHELXL package. The structures **FJI-H9** and **FJI-H10** were refined by full matrix least-squares with anisotropic displacement parameters for non-hydrogen atoms. All hydrogen atoms were generated geometrically and treated as riding. The crystallographic data are summarized in Table S1-S3. CCDC 1047502 and 1047503 contain the supplementary crystallographic data for **FJI-H9** and **FJI-H10**. These data can be obtained free of charge from The Cambridge Crystallographic Data Centre via www.ccdc.cam.ac.uk/data_request/cif.

Table S1 Crystal data and refinement results for compounds **FJI-H9** and **FJI-H10**.

Formula	$C_{24}H_{33}Ca_2N_3O_{12}S_2$ (**FJI-H9**)	$C_{10}H_{13}CaNO_6S$ (**FJI-H10**)
Formula weight	699.82	315.36
Crystal system	orthorhombic	monoclinic
space group	$Pca2_1$	$P2_1/n$
a (Å)	22.6505 (6)	6.5562 (2)
b (Å)	11.2352 (3)	10.6424 (5)
c (Å)	12.7961 (3)	18.2954 (6)
α (°)	90	90
β (°)	90	98.07 (3)
γ (°)	90	90
Volume ($Å^3$)	3256.39	1263.88
T (K)	100	100
Z	4	4
F (000)	1460	656
R1 (I>2□(I))	0.0411	0.0374
wR2 (reflections)	0.0957	0.0880
Goodness of fit on F2	1.075	1.065

Table S2 Selected Bond Lengths (Å) and Angle (º) for compound **FJI-H9**.

Bond	(Å)	Bond	(Å)
Ca1—O8^{i}	2.317 (3)	Ca2—O1^{iv}	2.292 (3)
Ca1—O11	2.338 (3)	Ca2—O5	2.333 (3)
Ca1—O4	2.346 (3)	Ca2—O9^{v}	2.386 (3)
Ca1—O2^{ii}	2.396 (3)	Ca2—O7^{vi}	2.407 (3)
Ca1—O6	2.460 (3)	Ca2—O10	2.417 (3)
Ca1—O9	2.507 (3)	Ca2—O4	2.537 (3)
Ca1—O10	2.538 (3)	Ca2—O3	2.544 (3)
		Ca2—O8^{vi}	2.647 (3)
Angle	(º)	Angle	(º)
O8^{i}—Ca1—O11	108.03 (12)	O1^{iv}—Ca2—O5	99.79 (13)
O8^{i}—Ca1—O4	164.47 (12)	O1^{iv}—Ca2—O9^{v}	81.41 (11)
O11—Ca1—O4	87.21 (12)	O5—Ca2—O9^{v}	82.93 (11)
O8^{i}—Ca1—O2^{ii}	88.55 (11)	O1^{iv}—Ca2—O7^{vi}	94.07 (13)
O11—Ca1—O2^{ii}	82.78 (12)	O5—Ca2—O7^{vi}	151.40 (12)
O4—Ca1—O2^{ii}	90.45 (11)	O9^{v}—Ca2—O7^{vi}	124.08 (11)
O8^{i}—Ca1—O6	76.68 (11)	O1^{iv}—Ca2—O10	82.23 (11)
O11—Ca1—O6	77.31 (11)	O5—Ca2—O10	77.68 (11)
O4—Ca1—O6	110.60 (11)	O9^{v}—Ca2—O10	152.02 (10)
O2^{ii}—Ca1—O6	149.95 (12)	O7^{vi}—Ca2—O10	79.60 (11)
O8^{i}—Ca1—O9	76.31 (11)	O1^{iv}—Ca2—O4	155.29 (11)
O11—Ca1—O9	164.62 (11)	O5—Ca2—O4	74.91 (12)
O4—Ca1—O9	88.19 (11)	O9^{v}—Ca2—O4	120.94 (10)
O2^{ii}—Ca1—O9	82.60 (11)	O7^{vi}—Ca2—O4	82.04 (11)
O6—Ca1—O9	118.01 (11)	O10—Ca2—O4	73.07 (10)
O8^{i}—Ca1—O10	94.93 (11)	O1^{iv}—Ca2—O3	152.73 (11)
O11—Ca1—O10	139.98 (11)	O5—Ca2—O3	93.79 (12)
O4—Ca1—O10	74.21 (10)	O9^{v}—Ca2—O3	76.90 (10)
O2^{ii}—Ca1—O10	131.28 (11)	O7^{vi}—Ca2—O3	84.71 (12)
O6—Ca1—O10	76.72 (10)	O10—Ca2—O3	124.00 (10)
O9—Ca1—O10	51.68 (9)	O4—Ca2—O3	51.57 (9)
		O1^{iv}—Ca2—O8^{vi}	80.15 (12)
		O5—Ca2—O8^{vi}	155.23 (11)
		O9^{v}—Ca2—O8^{vi}	72.53 (10)
		O7^{vi}—Ca2—O8^{vi}	51.96 (10)
		O10—Ca2—O8^{vi}	126.39 (10)

Symmetry codes: (i) 1-x, 2-y, -1/2+z; (ii) 1/2-x, y, -1/2+z; (iii) 1-x, 1-y, -1/2+z; (iv) 1/2+x, 1-y, z; (v) 1-x, 1-y, 1/2+z; (vi) x, -1+y, z; (vii) x, 1+y, z; (viii) 1-x, 2-y, 1/2+z; (ix) -1/2+x, 1-y, z; (x) 1/2-x, y, 1/2+z.

Table S3 Selected Bond Lengths (Å) and Angle (º) for compound **FJI-H10**.

Bond	(Å)
Ca1—O3^{i}	2.2889 (15)
Ca1—O6	2.335 (2)
Ca1—O4^{ii}	2.3352 (15)
Ca1—O5	2.3659 (16)
Ca1—O1	2.3732 (16)
Ca1—O4^{iii}	2.4343 (18)
Angle	(º)
O6—Ca1—O4^{ii}	81.29 (6)
O6—Ca1—O5	115.69 (6)
O4^{ii}—Ca1—O5	75.08 (6)
O6—Ca1—O1	89.24 (7)
O4^{ii}—Ca1—O1	128.80 (6)
O5—Ca1—O1	149.50 (6)
O6—Ca1—O4^{iii}	137.07 (6)
O4^{ii}—Ca1—O4^{iii}	73.09 (6)
O5—Ca1—O4^{iii}	90.50 (6)
O1—Ca1—O4^{iii}	81.17 (6)

Symmetry codes: (i) 1/2+x, 3/2-y, 1/2+z; (ii) -1/2+x, 3/2-y, 1/2+z; (iii) 1/2-x, -1/2+y, 1/2-z; (iv) -x, 1-y, 1-z; (v) 1-x, 1-y, 1-z; (vi) -1/2+x, 3/2-y, -1/2+z; (vii) 1/2-x, 1/2+y, 1/2-z; (viii) 1/2+x, 3/2-y, -1/2+z.

Heavy metal ions adsorption test of FJI-H9

Inside a capped 25-mL vial, fresh crystals of **FJI-H9** were allowed to soak undisturbed in 0.1 mol/L acetonitrile solution of Cd(II), Hg(II), Ni(II), Mn(II), Zn(II), Fe(II), Pb(II), Co(II) and Mg(II) for 15 hours. The mixture were then filtered, and the solids that was isolated were then washed three times with 30 mL of acetonitrile to give final products. ICP elemental analysis revealed that the weight percentage ratios of M/Ca in compounds **FJI-H9**.

Table S4 The weight ratios of M/Ca in **FJI-H9**.

	Cd^{2+}	Hg^{2+}	Ni^{2+}	Mn^{2+}	Zn^{2+}	Fe^{2+}	Pb^{2+}	Co^{2+}	Mg^{2+}
M/Ca	2.50	0.34	0.08	0.07	0.06	0.07	0.009	0.07	0.03

Heavy metal ions adsorption test of FJI-H10

Inside a capped 25-mL vial, fresh crystals of **FJI-H10** were allowed to soak undisturbed in 0.1 mol/L acetonitrile solution of Cd(II), Hg(II) for 15 hours. The mixture were then filtered, and the solids that was isolated were then washed three times with 30 mL of acetonitrile to give final products. ICP elemental analysis revealed that the weight percentage ratios of M/Ca in compounds **FJI-H10**.

Table S5 The weight ratios of M/Ca in **FJI-H10**.

	Cd^{2+}	Hg^{2+}
M/Ca	**0.001**	**0.28**

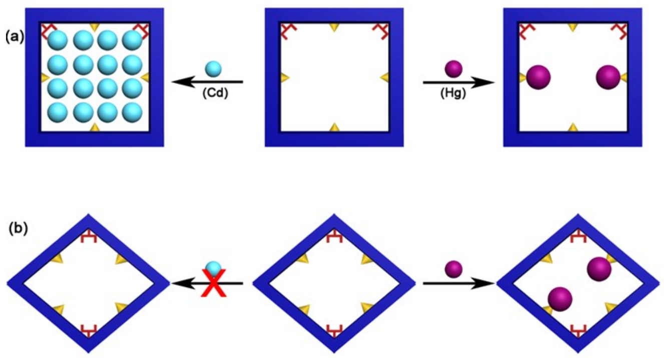

Scheme 2 Possible adsorption mechanisms: (a) with help by DMA molecules highlighted as red fork and confined cavity, the Cd(II) ions closing pack in the square channel of **FJI-H9**; only part of thiophenyl groups highlighted as yellow triangle of **FJI-H9** adsorb Hg(II). (b) nearly no Cd(II) can be absorbed by **FJI-H10**, only part of thiophenyl groups highlighted as yellow triangle of **FJI-H10** adsorb Hg(II).

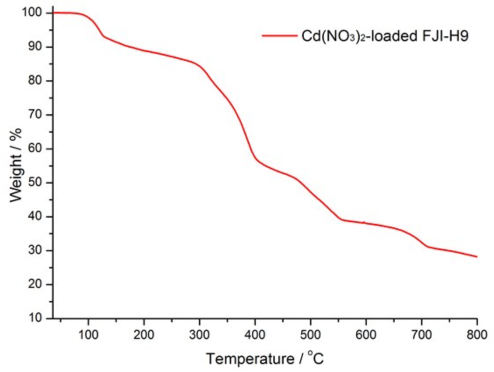

Figure S3 TGA curves. The sample of **FJI-H9-$Cd(NO_3)_2$**, about 15.5% volatile solvents.

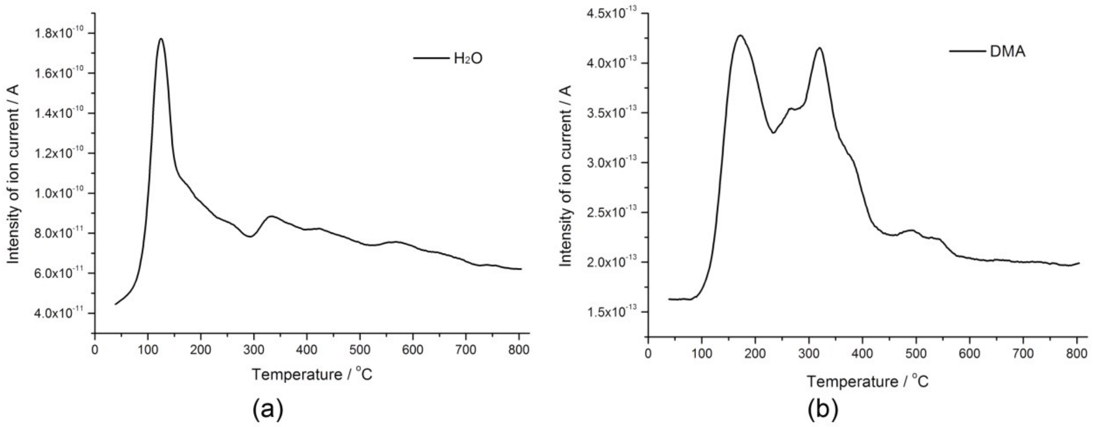

Figure S4 Thermogravimetry-Mass curves. (a): H_2O; (b): DMA.

The sorption isotherms of Cd^{2+}

The adsorption capacity of the **FJI-H9** adsorbent was investigated with a Cd^{2+} solution at concentration from 10 to 250 ppm. The uptake of Cd^{2+} by the adsorbent can be described in the form of sorption isotherms, which delineates the adsorption capacity (the amount of guest adsorbed divided by the sorbent mass) as a function of the guest concentration in the solution (Fig. S5). The amount of adsorption at equilibrium q_e (mg/g), was calculated according to eqn.

$$q_e = (c_0 - c_e)v/m$$

where c_0 (mg/L) and c_e (mg/L) are the initial and equilibrated concentrations of Cd^{2+} respectively, v (L) is the volume of the solution, and m (g) is the mass of the adsorbent.

The existence of a peak indicates the final saturation of this adsorbent, giving an adsorption capacity of *ca*. 225 mg g^{-1} when the initial Cd^{2+} concentration was 150 ppm. Such a high adsorption of Cd(II) by **FJI-H9** may results from an unusual synergy from active sites and confined cavity.

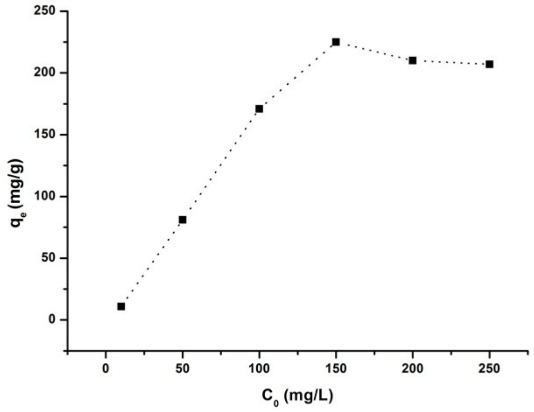

Figure S5 Sorption isotherm of Cd^{2+} by **FJI-H9**. (v = 30 mL, m (adsorbent) = 10 mg, T = 20 °C, t = 15 h).

The selective adsorption of FJI-H9 under background metal ions

Further research we also studied the selective adsorption of **FJI-H9** on various metal ions (Ca^{2+}, Mg^{2+}, Co^{2+}, Ni^{2+}, Mn^{2+}, Zn^{2+}, Fe^{2+}, Pb^{2+}, Hg^{2+}, Cd^{2+}). Excessive **FJI-H9** crystals were put into the solution, which contained ten different kinds of metal ions and the initial concentrations were 100 ppm. As shown in Figure S6, **FJI-H9** can remain effective in the presence of low concentrations of these background metal ions, Cd(II) ions can be effectively adsorbed while Hg(II) can be moderated adsorbed. In contrast, other background metal ions such as Ca(II), Mg(II), Co(II), Ni(II), Mn(II), Zn(II), Fe(II) and Pb(II) do not quite bind to **FJI-H9**.

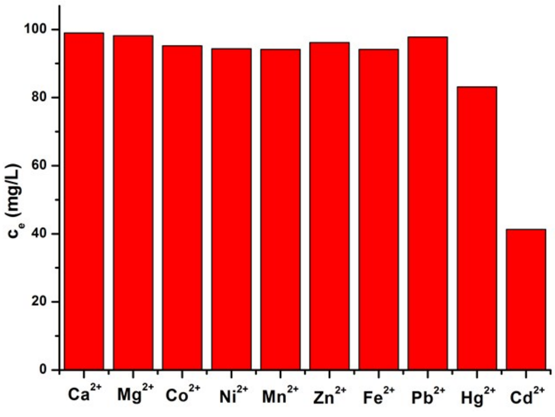

Figure S6 The selective adsorption of Cd^{2+} by **FJI-H9**.

EXAFS measurements

EXAFS measurements at Cd K-edge were BL14W1 station in SSRF and 1W1B station in BSRF. The storage rings of NSRL, SSRF, and BSRF were operated at 0.8 GeV with the current of 250 mA, at 3.5 GeV with the current of 300 mA, and at 2.5 GeV with a maximum current of 250 mA, respectively. The acquired EXAFS data were processed according to the standard procedures using the ATHENA module implemented in the IFEFFIT software packages. The k^3-weighted EXAFS spectra were obtained by subtracting the post-edge background from the overall absorption and then normalizing with respect to the edge-jump step. Subsequently, k^3-weighted $\chi(k)$ data in the k-space ranging from 2.4–14.0 Å^{-1} were Fourier transformed to real (R) space using a hanning windows (dk = 1.0 Å^{-1}) to separate the EXAFS contributions from different coordination shells.

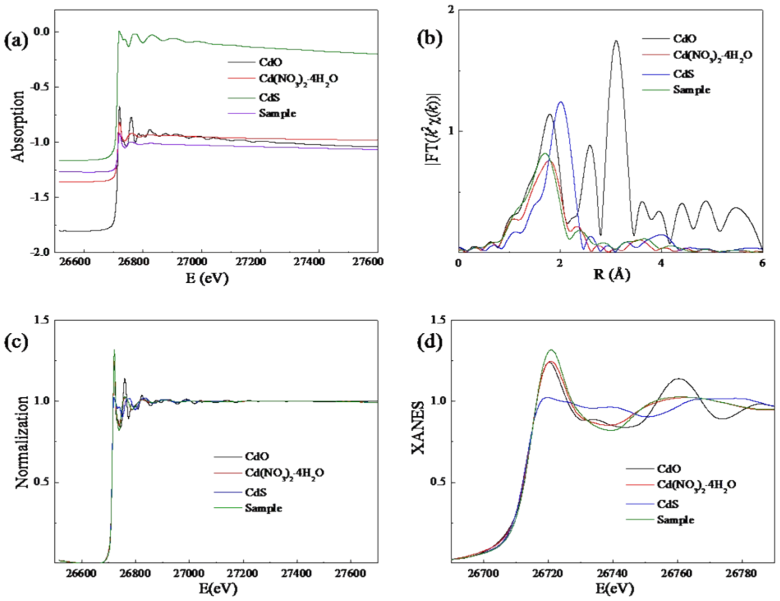

Figure S7 EXAFS fitting details: (a) adsorption; (b) the corresponding Fourier transforms FT(k^{3}w(k)); (c) normalization; (d) XANES.

Desorption of FJI-H9

The sample (10 mg), which adsorbed $Cd(NO_3)_2$ was added to acetonitrile-EDA (v:v = 25:1) solution, and this solution was undisturbed for 24 hours. The mixture was then filtered, and the solid that was isolated was then washed three times with 30 mL of

acetonitrile to give a final product (9 mg). The ensuing ICP elemental analysis yielded a Cd/Ca ratio of 0.003:1, which indicated that about 99.9% of $Cd(NO_3)_2$ components had been removed from the solid sample.

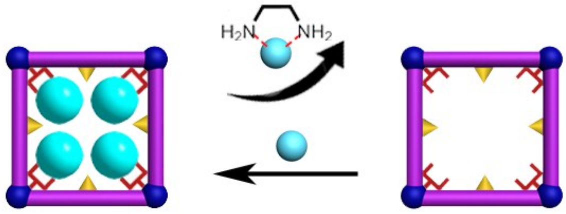

Scheme 3 Desorption and resorption of $Cd(NO_3)_2$ by **FJI-H9**.

Secondary adsorption of $Cd(NO_3)_2$ test

$Cd(NO_3)_2$-depleted sample of **FJI-H9** was once again added in the 0.1 mol/L acetonitrile solution of $Cd(NO_3)_2$ for 15 hours, then powder XRD showed the main framework remained intact, and ICP revealed the weight ratio of Cd/Ca reduced to 2.03:1.

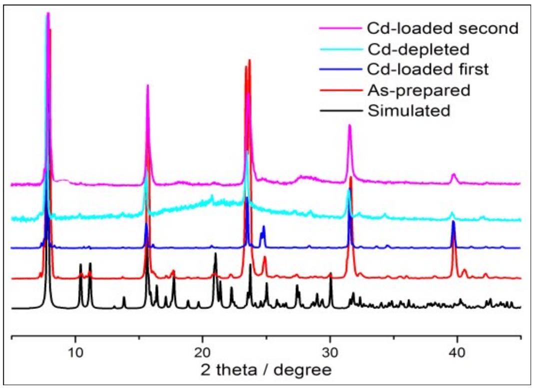

Figure S8 Powder X-ray diffraction patterns. Black: a simulation sample of **FJI-H9**; red: a synthesized sample of **FJI-H9**; blue: a first $Cd(NO_3)_2$-loaded sample of **FJI-H9**; light blue: a $Cd(NO_3)_2$-depleted sample of **FJI-H9-Cd(NO$_3$)$_2$**; purple: a second $Cd(NO_3)_2$-loaded sample of **FJI-H9**.

Table S6 The weight ratios of Cd/Ca in **FJI-H9** for the first and second times.

	First time	Second time
Cd/Ca	2.50	2.03

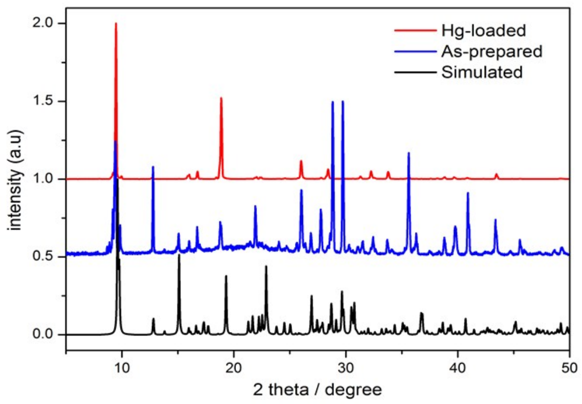

Figure S9 Powder X-ray diffraction patterns. Black: a simulation sample of **FJI-H10**; blue: a synthesized sample of **FJI-H9**; red: a $Hg(NO_3)_2$-loaded sample of **FJI-H10.**

Regeneration used FJI-H9 into fresh one

The 10 mg used **FJI-H9** powder and 50 uL HNO_3 (about 16 mol/L) were added to 5 mL mother liquor which has been used to prepared fresh **FJI-H9**, heating such solution at 85 ^{o}C for six days leads to 9 mg fresh **FJI-H9** crystal (yield: 90%).

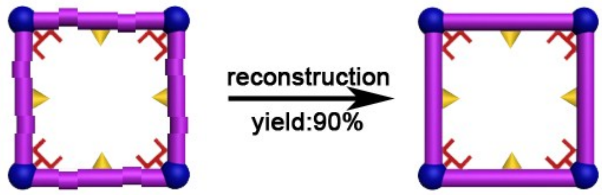

Scheme 4 *In situ* reconstruction of the used framework into fresh framework.

Fluorescence indicator

We put **FJI-H9** into various solutions of Cd^{2+} with gradually increasing concentration from 10 ppm to 200 ppm for 10 minutes. The intensity slight reduced when the concentration gradually increased from 10 ppm to 200 ppm. Even more important, soaking **FJI-H9** in the 10 ppm solution of Cd^{2+} for 10 minutes had already led to highly quench their emission (λem = 460 nm). In contrast, for other metal ions such as Hg(II), Mg(II), Co(II), Ni(II), Mn(II), Zn(II), Fe(II) and Pb(II), no such obvious

fluorescence quenching can be observed under the same condition.

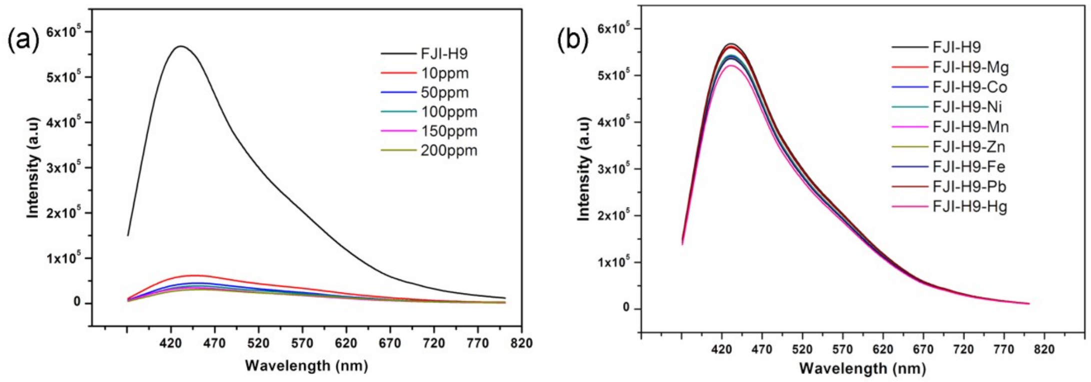

Figure S10 (a) Rapid detection of cadmium with low concentration; (b) **FJI-H9** respond to other metal ions.
